# Supplementary material for: Convergent Validation of a Self-Reported Commuting to and from School Diary in Spanish Adolescents
Source: Int J Environ Res Public Health. 2022 Dec 20;20(1):18. doi: 10.3390/ijerph20010018 (PMC9819640; doi:10.3390/ijerph20010018)
Supplement: Supplementary file 1 [file ijerph-20-00018-s001.zip › Supplementary Material S1.pdf]

SUBJECT ID: **FECHA:**

DAY 1	
Time I get up from bed	
Time I leave home	
Time I arrive at school	
Time I leave school	
Time I arrive at home	
Time I take off my belt	From:<br>To:
Time I take off my belt	From:<br>To:
Time I go to bed	

DAY 2	
Time I get up from bed	
Time I leave home	
Time I arrive at school	
Time I leave school	
Time I arrive at home	
Time I take off my belt	From:<br>To:
Time I take off my belt	From:<br>To:
Time I go to bed	

DAY 3	
Time I get up from bed	
Time I leave home	
Time I arrive at school	
Time I leave school	
Time I arrive at home	
Time I take off my belt	From:<br>To:
Time I take off my belt	From:<br>To:
Time I go to bed	

DAY 4	
Time I get up from bed	
Time I leave home	
Time I arrive at school	
Time I leave school	
Time I arrive at home	
Time I take off my belt	From:<br>To:
Time I take off my belt	From:<br>To:
Time I go to bed	

DAY 5	
Time I get up from bed	
Time I leave home	
Time I arrive at school	
Time I leave school	
Time I arrive at home	
Time I take off my belt	From:<br>To:
Time I take off my belt	From:<br>To:
Time I go to bed	

DAY 6	
Time I get up from bed	
Time I leave home	
Time I arrive at school	
Time I leave school	
Time I take off my belt	
Time I take off my belt	From:<br>To:
Time I go to bed	From:<br>To:
Time I take off my belt	

DAY 7	
Time I get up from bed	
Time I leave home	
Time I arrive at school	
Time I leave school	
Time I arrive at home	
Time I take off my belt	From:<br>To:
Time I take off my belt	From:<br>To:
Time I go to bed	

ID DE SUJETO: **FECHA:**

DÍA 1	
Hora a la que me levanto	
Hora a la que salgo de casa	
Hora a la que llego al IES	
Hora a la que salgo de IES	
Hora a la que llego a casa	
Hora a la que me quito cinturón	Desde:<br>Hasta:
Hora a la que me quito cinturón	Desde:<br>Hasta:
Hora a la que me acuesto	

DÍA 2	
Hora a la que me levanto	
Hora a la que salgo de casa	
Hora a la que llego al IES	
Hora a la que salgo de IES	
Hora a la que llego a casa	
Hora a la que me quito cinturón	Desde:<br>Hasta:
Hora a la que me quito cinturón	Desde:<br>Hasta:
Hora a la que me acuesto	

DÍA 3	
Hora a la que me levanto	
Hora a la que salgo de casa	
Hora a la que llego al IES	
Hora a la que salgo de IES	
Hora a la que llego a casa	
Hora a la que me quito cinturón	Desde:<br>Hasta:
Hora a la que me quito cinturón	Desde:<br>Hasta:
Hora a la que me acuesto	

DÍA 4	
Hora a la que me levanto	
Hora a la que salgo de casa	
Hora a la que llego al IES	
Hora a la que salgo de IES	
Hora a la que llego a casa	
Hora a la que me quito cinturón	Desde:<br>Hasta:
Hora a la que me quito cinturón	Desde:<br>Hasta:
Hora a la que me acuesto	

DÍA 5	
Hora a la que me levanto	
Hora a la que salgo de casa	
Hora a la que llego al IES	
Hora a la que salgo de IES	
Hora a la que llego a casa	
Hora a la que me quito cinturón	Desde:<br>Hasta:
Hora a la que me quito cinturón	Desde:<br>Hasta:
Hora a la que me acuesto	

DÍA 6	
Hora a la que me levanto	
Hora a la que salgo de casa	
Hora a la que llego al IES	
Hora a la que salgo de IES	
Hora a la que llego a casa	
Hora a la que me quito cinturón	Desde:<br>Hasta:
Hora a la que me quito cinturón	Desde:<br>Hasta:
Hora a la que me acuesto	

DÍA 7	
Hora a la que me levanto	
Hora a la que salgo de casa	
Hora a la que llego al IES	
Hora a la que salgo de IES	
Hora a la que llego a casa	
Hora a la que me quito cinturón	Desde:<br>Hasta:
Hora a la que me quito cinturón	Desde:<br>Hasta:
Hora a la que me acuesto	
