# Supplementary material for: Convergent Validation of a Self-Reported Commuting to and from School Diary in Spanish Adolescents
Source: Int J Environ Res Public Health. 2022 Dec 20;20(1):18. doi: 10.3390/ijerph20010018 (PMC9819640; doi:10.3390/ijerph20010018)
Supplement: Supplementary file 1 [file ijerph-20-00018-s001.zip › Supplementary Material S2.pdf]

Supplementary Material S2. Self-reported diary time versus objective time in the total commuting time, by gender.

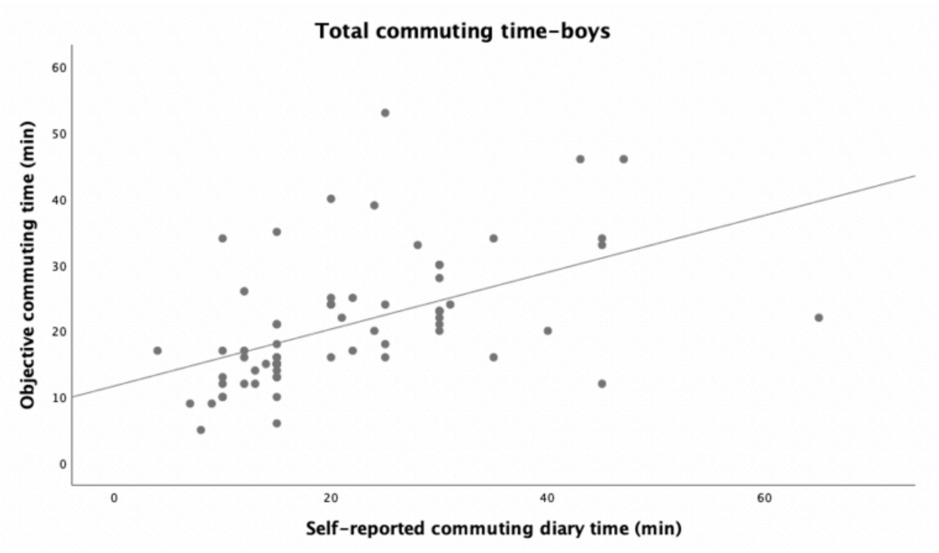

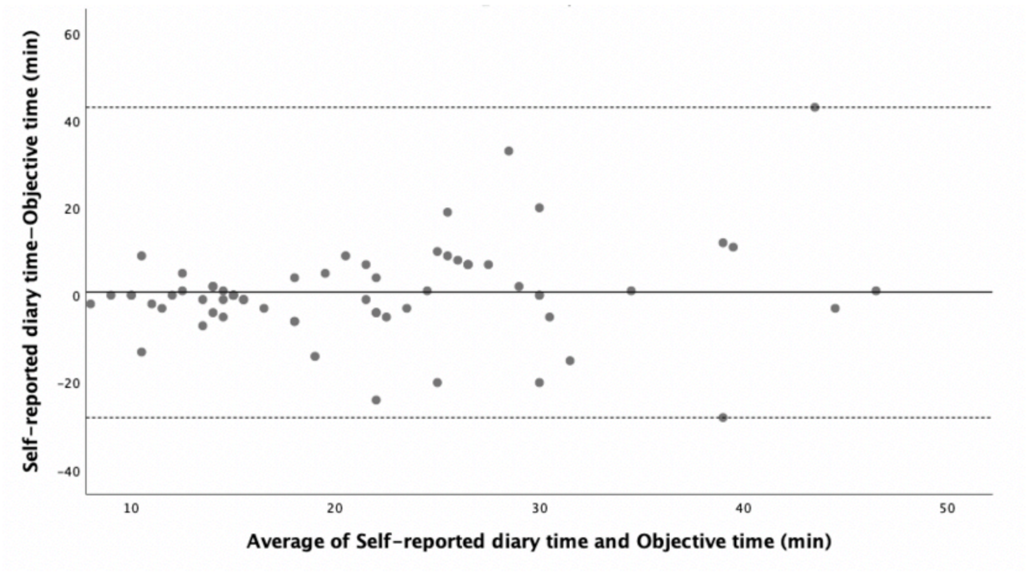

Figure S1. Commuting time expressed as minutes (total commuting time-boys). (a) Regression of the objective commuting time (y) vs. (x) the self-reported commuting diary time. The regression equation is y=11.66 + 0.43x. (b) Bland–Altman plot of the total commuting time between self-reported diary time and objective time (y) vs. Average of self-reported diary time and objective time (x). The central dotted line represents the mean of differences between the self-report time measure and the objective time measure; the upper and lower dotted lines represent the upper and lower 95% limits of agreement (mean difference±1.96 standard deviations of the differences), respectively.

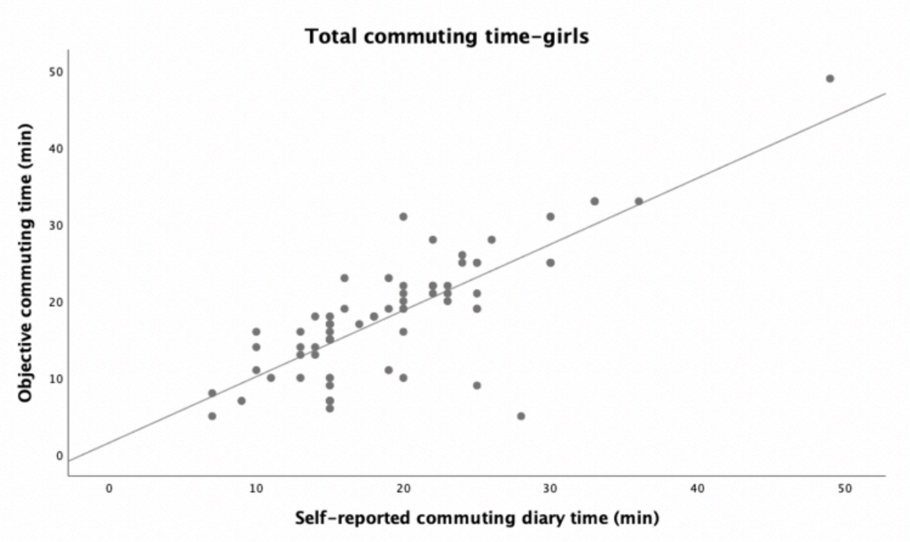

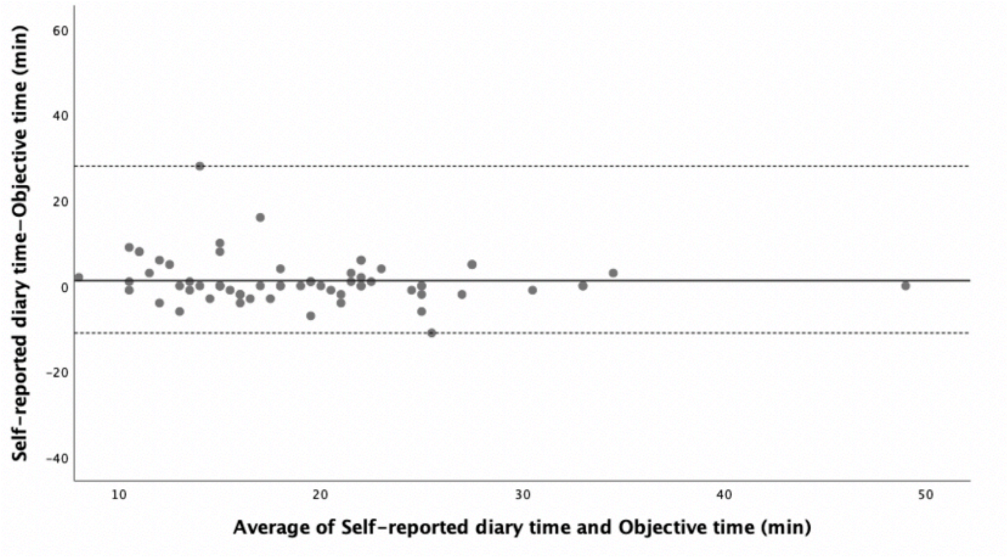

Figure S2. Commuting time expressed as minutes (total commuting time-girls). (a) Regression of the objective commuting time (y) vs. (x) the self-reported commuting diary time. The regression equation is y=1.54 + 0.86x. (b) Bland–Altman plot of the total commuting time between self-reported diary time and objective time (y) vs. Average of self-reported diary time and objective time (x). The central dotted line represents the mean of differences between the self-report time measure and the objective time measure; the upper and lower dotted lines represent the upper and lower 95% limits of agreement (mean difference±1.96 standard deviations of the differences), respectively.
