# Supplementary material for: Convergent Validation of a Self-Reported Commuting to and from School Diary in Spanish Adolescents
Source: Int J Environ Res Public Health. 2022 Dec 20;20(1):18. doi: 10.3390/ijerph20010018 (PMC9819640; doi:10.3390/ijerph20010018)
Supplement: Supplementary file 1 [file ijerph-20-00018-s001.zip › Supplementary Material S3.pdf]

Supplementary Material S3. Self-reported diary time versus objective time in the home-school and school-home trips, for starting and ending time.

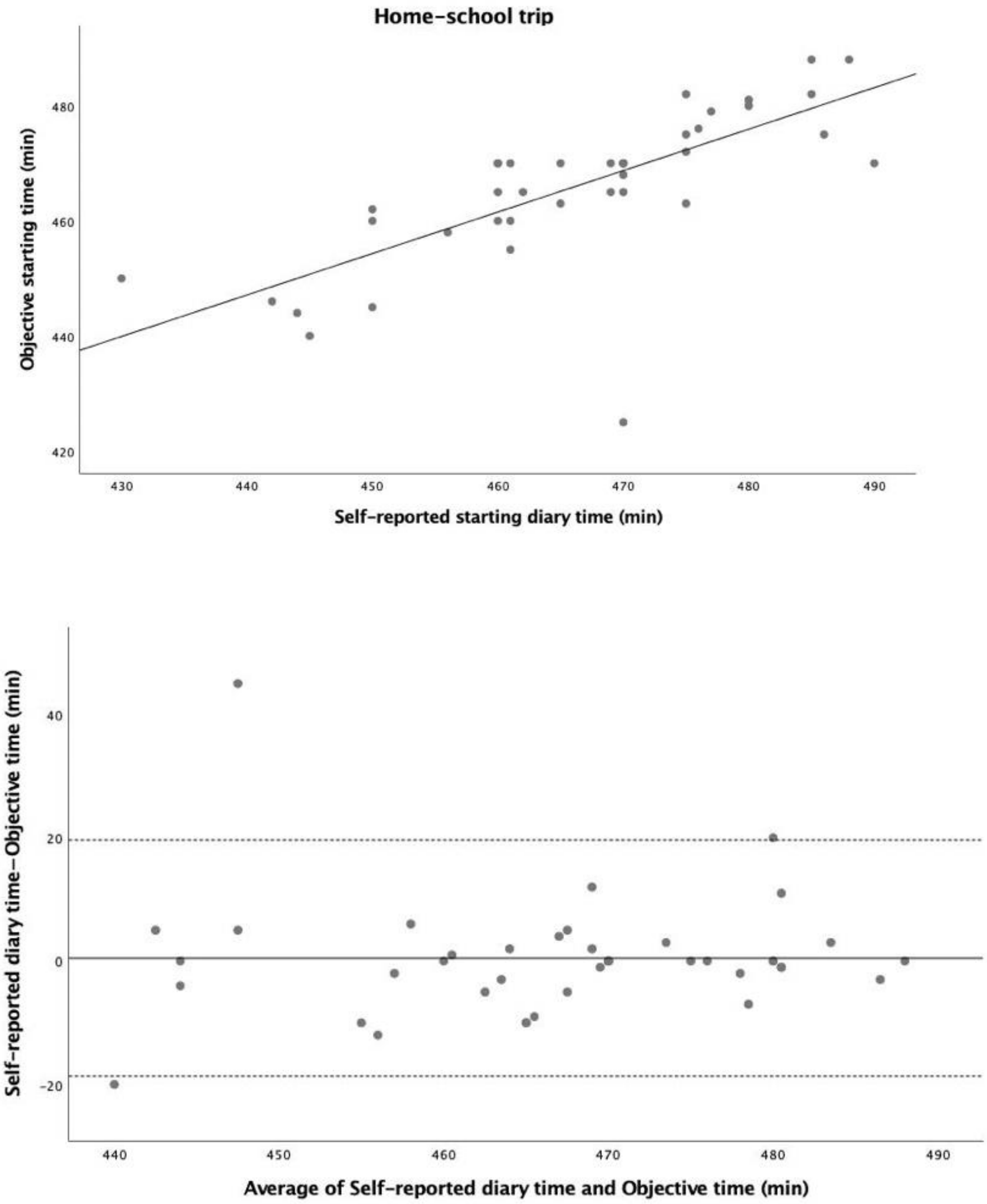

Figure S3. Starting time expressed as minutes (**home-school trip**). (a) Regression of the objective commuting time (y) vs. (x) the self-reported commuting diary time. The regression equation is y=1.3 + 0.72x, r 0.735, P< 0.001. (b) Bland–Altman plot of the starting time of the **home-school trip** between self-reported diary time and objective time (y) vs. Average of self-reported diary time and objective time (x). The central dotted line represents the mean of differences between the objective time measure and the self-report time measure; the upper and lower dotted lines represent the upper and lower 95% limits of agreement (mean differences+1.96 standard deviations of the differences), respectively. Adolescents reported 0.4 minutes (or 24 s) more in the home-school trip starting time than the objective time (95% limits of agreement were 19.6 min and −18.7 min).

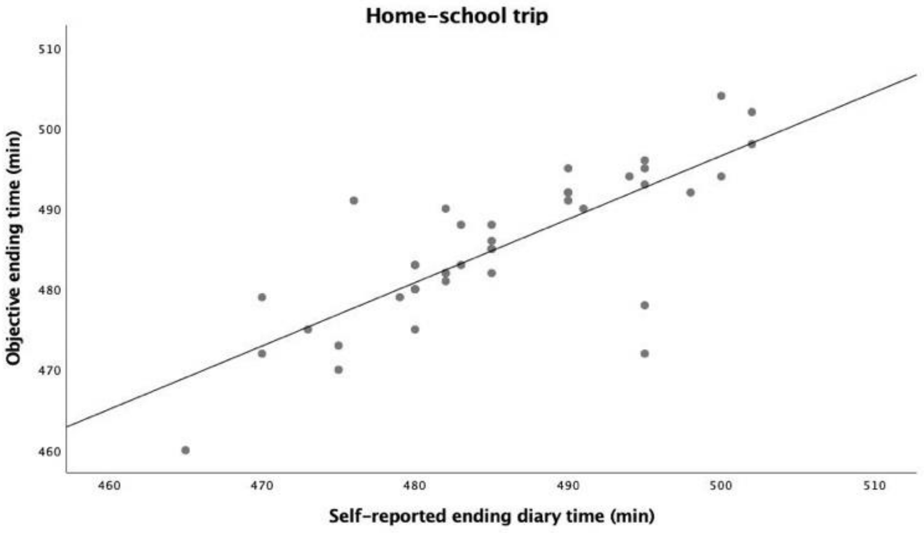

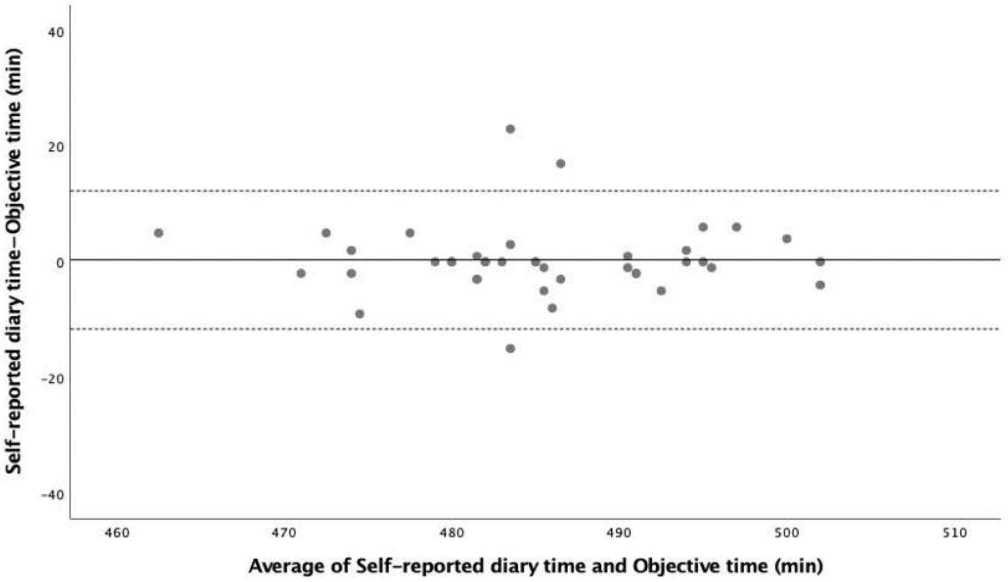

Figure S4. Ending time expressed as minutes (**home-school trip**). (a) Regression of the objective commuting time (y) vs. (x) the self-reported commuting diary time. The regression equation is y=1.03 + 0.79x, r 0.784, P< 0.001. (b) Bland–Altman plot of the ending time of the **home-school trip** between self-reported diary time and objective time (y) vs. Average of self-reported diary time and objective time (x). The central dotted line represents the mean of differences between the objective time measure and the self-report time measure; the upper and lower dotted lines represent the upper and lower 95% limits of agreement (mean differences+1.96 standard deviations of the differences), respectively. Adolescents reported 0.3 minutes (or 18 s) more in the home-school trip ending time than the objective time (95% limits of agreement were 12.3 min and −11.6 min).

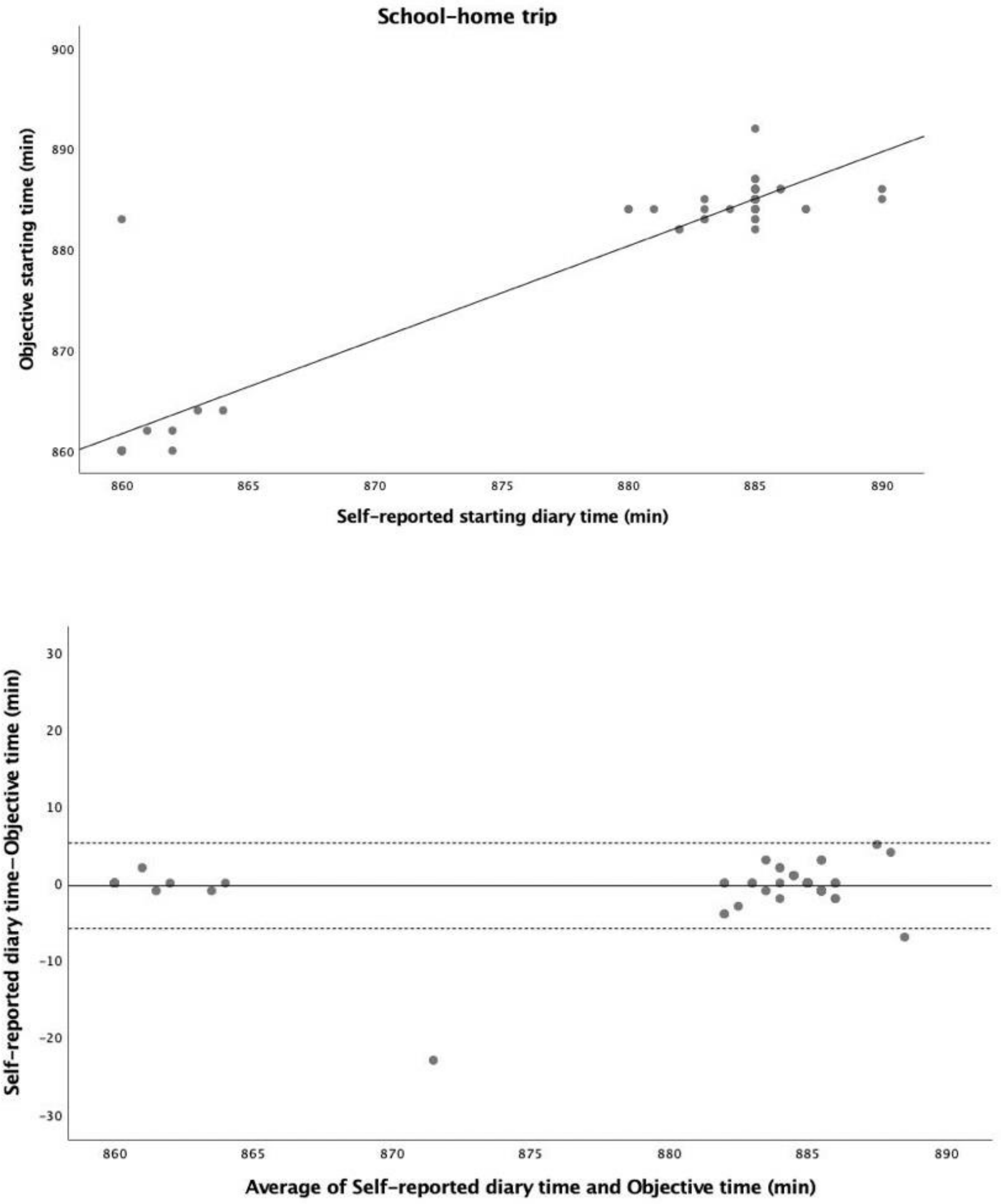

Figure S5. Starting time expressed as minutes (**school-home trip**). (a) Regression of the objective commuting time (y) vs. (x) the self-reported commuting diary time. The regression equation is y=58.62 + 0.93x, r 0.956, P< 0.001. (b) Bland–Altman plot of the starting time of the **school-home trip** between self-reported diary time and objective time (y) vs. Average of self-reported diary time and objective time (x). The central dotted line represents the mean of differences between the objective time measure and the self-report time measure; the upper and lower dotted lines represent the upper and lower 95% limits of agreement (mean differences+1.96 standard deviations of the differences), respectively. Adolescents reported 0.3 minutes (or 18 s) less in the school-home trip starting time than the objective time (95% limits of agreement were 5.2 min and −5.9 min).

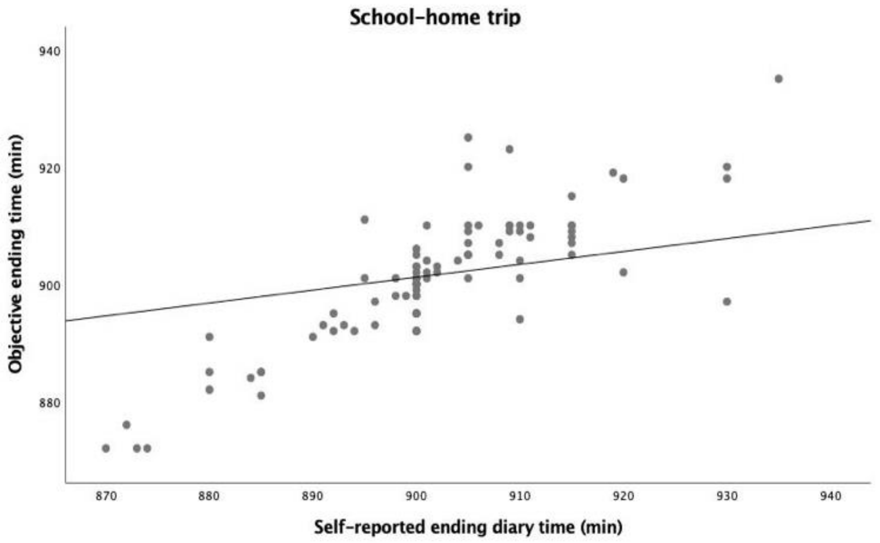

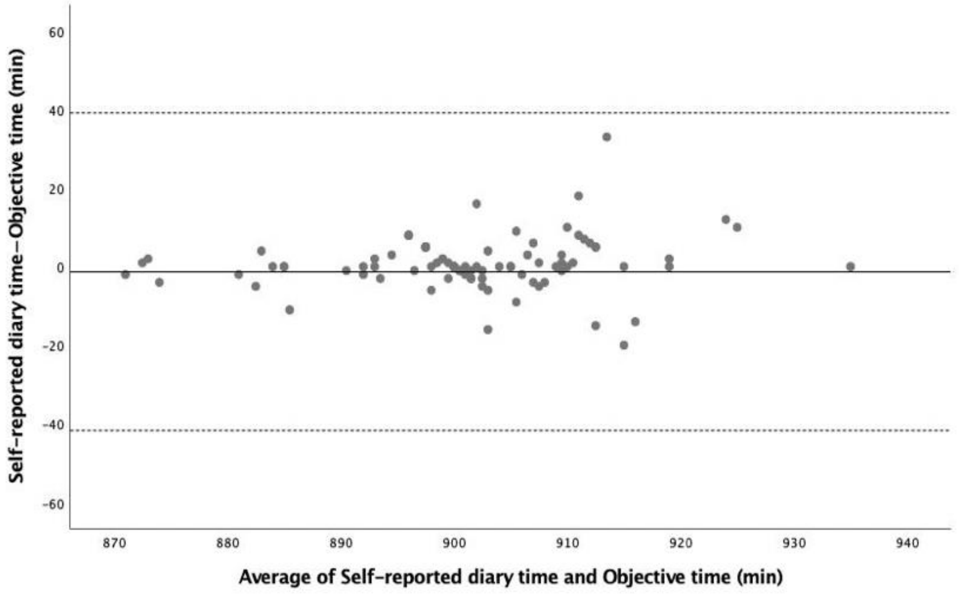

Figure S6. Ending time expressed as minutes (**school-home trip**). ((a) Regression of the objective commuting time (y) vs. (x) the self-reported commuting diary time. The regression equation is y=7.03 + 0.22x, r 0.450, P< 0.001. (b) Bland–Altman plot of the ending time of the **school-home trip** between self-reported diary time and objective time (y) vs. Average of self-reported diary time and objective time (x). The central dotted line represents the mean of differences between the objective time measure and the self-report time measure; the upper and lower dotted lines represent the upper and lower 95% limits of agreement (mean differences+1.96 standard deviations of the differences), respectively. Adolescents reported 1.3 minutes less in the school-home trip ending time than the objective time (95% limits of agreement were 39.1 min and −41.7 min).
